# Crystal Structure of the Neutralizing Llama V_HH_ D7 and Its Mode of HIV-1 gp120 Interaction

**DOI:** 10.1371/journal.pone.0010482

**Published:** 2010-05-05

**Authors:** Andreas Hinz, David Lutje Hulsik, Anna Forsman, Willie Wee-Lee Koh, Hassan Belrhali, Andrea Gorlani, Hans de Haard, Robin A. Weiss, Theo Verrips, Winfried Weissenhorn

**Affiliations:** 1 Unit of Virus Host Cell Interactions (UVHCI), UMI 3265, Université Joseph Fourier-EMBL-CNRS, Grenoble, France; 2 Department of Cellular Architecture and Dynamics, University of Utrecht, Utrecht, The Netherlands; 3 Division of Infection and Immunity, MRC/UCL Centre for Medical Molecular Virology, University College London, London, United Kingdom; 4 European Molecular Biology Laboratory, Grenoble, France; Institut Pasteur, France

## Abstract

HIV-1 entry into host cells is mediated by the sequential binding of the envelope glycoprotein gp120 to CD4 and a chemokine receptor. Antibodies binding to epitopes overlapping the CD4-binding site on gp120 are potent inhibitors of HIV entry, such as the llama heavy chain antibody fragment V_HH_ D7, which has cross-clade neutralizing properties and competes with CD4 and mAb b12 for high affinity binding to gp120. We report the crystal structure of the D7 V_HH_ at 1.5 Å resolution, which reveals the molecular details of the complementarity determining regions (CDR) and substantial flexibility of CDR3 that could facilitate an induced fit interaction with gp120. Structural comparison of CDRs from other CD4 binding site antibodies suggests diverse modes of interaction. Mutational analysis identified CDR3 as a key component of gp120 interaction as determined by surface plasmon resonance. A decrease in affinity is directly coupled to the neutralization efficiency since mutations that decrease gp120 interaction increase the IC50 required for HIV-1 IIIB neutralization. Thus the structural study identifies the long CDR3 of D7 as the key determinant of interaction and HIV-1 neutralization. Furthermore, our data confirm that the structural plasticity of gp120 can accommodate multiple modes of antibody binding within the CD4 binding site.

## Introduction

The envelope glycoprotein (Env) from the human immunodeficiency virus type 1 (HIV-1) forms a heterotrimer composed of the receptor binding subunit gp120 and the membrane anchored fusion protein subunit gp41. Entry into host cells is mediated by gp120 interaction with CD4 that triggers a conformational change allowing subsequent interaction with cellular coreceptors such as CCR5 or CXCR4 [1]–[4]. Together these events trigger a refolding of gp41 that leads to the fusion of virus and host cell membranes [5]–[7]. Env is the target for entry inhibitors [8] and neutralizing antibodies directed against gp120 and gp41 [9]. A main problem in HIV-1 vaccine research is the generation of cross-subtype neutralizing antibodies, which is due to the fact that HIV-1 employs a number of strategies to evade the immune response. This includes highly variable gp120 regions, a carbohydrate shield [10] and conformational masking of the receptor binding site [11]. The overall conformational flexibility of gp120 is highlighted by the differences between the native SIV gp120 core structure [12] and structures representing the CD4- and antibody-induced conformations of the HIV-1 gp120 [13]–[16]. Gp120 structures are composed of an inner and an outer domain; the inner domain varies substantially including the refolding of the bridging sheet, while the outer domain harbouring the CD4 binding site is mostly conserved except for the refolding of the CD4-binding loop [12], [13]. The conformational flexibility is considered to be the main obstacle to the development of an HIV-1 vaccine, besides the sequence variability and the glycan shield. Consequently, only few broadly neutralizing antibodies have been described to date [17]. MAbs 2F5, 4E10 and Z13 recognize epitopes within the membrane proximal region of gp41 [18]–[21], mAb 2G12 recognizes a carbohydrate motif [22], [23], b12 interacts within the CD4 binding site [24], [25], HJ16 overlaps with the CD4 binding site [26] and antibodies PG9 and PG16 are specific for the trimeric Env conformation [27].

The crystal structure of gp120 in complex with b12 revealed the molecular details including a substantial conserved gp120 surface overlapping between both the CD4- and b12-bound states [14]. The similarities of both interactions is highlighted by the fact that b12 employs Tyr^53^ to fill the hydrophobic pocket in gp120 that is otherwise occupied by CD4 Phe^43^ [14]. MAb b12 is broadly neutralizing since it engages gp120 at the same exposed surface in a similar manner as CD4, albeit it does not require the induction of further conformational changes [14]. The CD4 binding site is highly conserved, but nonetheless not all antibodies targeting the CD4 binding site show broad cross-clade neutralization properties including F105, M12 and M14 for example [15], [28]–[32]. No breakthrough has yet been reported regarding the efficient generation of broadly neutralizing monoclonal antibodies upon immunization of animals with Env antigens [33], [34] except for the generation of camelid antibodies. Three heavy chain only camelid specific antibody domains D7, A12 and C8, termed V_HH_, have been isolated after immunization with gp120. These antibodies compete with CD4 and b12 for gp120 interaction and exert neutralizing activity against primary isolates of subtypes B and C [35].

Here we describe the crystal structure of the camelid V_HH_ D7 and determine the molecular determinants for HIV-1 Env gp120 interaction. Mutagenesis of selected CDR residues abrogate or enhance gp120 interaction *in vitro* and correlate with the neutralization activity of D7 against the B-clade HIV-1 IIIB thus providing a molecular model for D7-gp120 reactivity.

## Results and Discussion

### Structure of the V_HH_ D7

**Figure 1:**
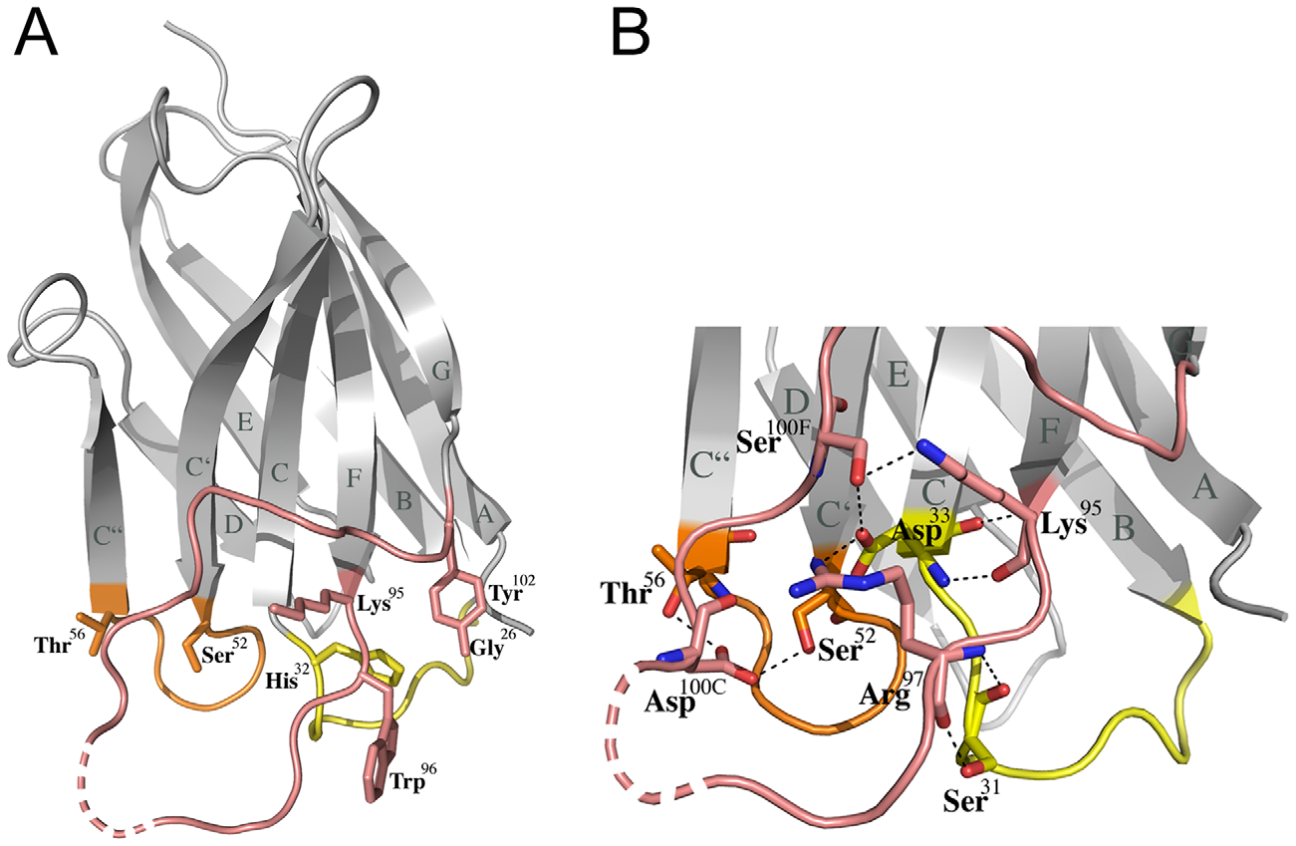
Structure of the llama heavy chain antibody fragment V_HH_ D7. (**A**) Ribbon representation of D7; the complementarity determining regions (CDR) are highlighted in yellow (CDR1), orange (CDR2) and salmon (CDR3). The first and last residue of each CDR is shown together with the side chain of Trp^96^ critical for gp120 interaction and neutralization. The dotted line indicates CDR3 residues lacking continuous main chain density for residues Arg^100^ to Ser^100B^. (**B**) A close-up of the CDR interaction network reveals multiple polar interactions between CDR1 and CDR3 as well as CDR2 and CDR3.

**Figure 2:**
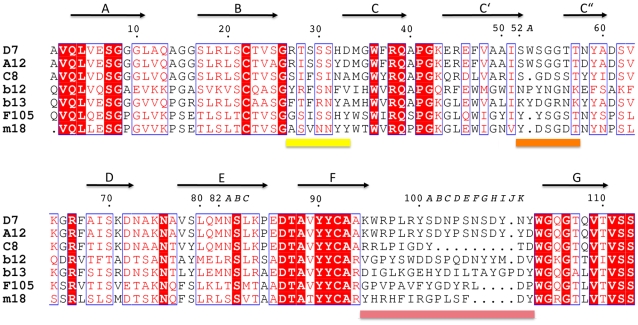
Structure based sequence alignment of D7 with V_HH_ A12 and C8 as well as with the V_H_ domains from the neutralizing antibodies b12, b13, F105 and m18. The residue numbering is according to Chothia [38] and the CDRs are indicated by coloured bars.

The crystal structure of the llama heavy chain antibody fragment V_HH_ D7 was solved by molecular replacement and refined to a resolution of 1.5 Å with an R factor of 16.6% and an R_free_ of 19.4% (table 1). D7 folds into a typical immunoglobulin domain closely resembling known llama V_HH_ structures [36] (Figure 1A). It contains two canonical (CDR1 and CDR2) and a long CDR3 typical for llama V_HH_s [37] with a non-canonical CDR conformation [38]. CDR3 is composed of 18 residues (Lys^95^ – Tyr^102^) (Figure 2). The base of CDR3 is well defined and stabilized by multiple main chain and side chain interactions including hydrogen bonds and salt bridges with CDR1 (Ser^31^-Arg^97^, Asp^33^-Lys^95^, Asp^33^-Arg^97^ and Asp^33^-Ser^100F^) and CDR2 (Ser^52^-Asp^100C^ and Thr^56^-Asp^100C^) (Figure 1B). The extensive inter CDR stabilization suggests a potentially lower flexibility of the CDRs upon binding to gp120. The CD4 binding site antibody b12 employs only one polar (Ser^30^-Tyr^53^) and few hydrophobic inter heavy chain CDR contacts [14], [39]. However, the tip of the D7 CDR3 (Arg^100^ - Ser^100B^) is highly mobile evidenced by the lack of continuous main chain density for three residues, including Tyr^100A^ positioned at the apex of CDR3, indicating that their conformational flexibility might be important for gp120 recognition.

**Table 1:** X-ray data collection and refinement statistics.

Unit cell dimensions	
a (Å)	37.37
b (Å)	62.18
c (Å)	62.74
Space group	P2_1_2_1_2_1_
Wavelength (Å)	0.974
Resolution (Å)	44.0-1.5
Completeness (%)	94.1 (69.7)
Total reflections	150510
Unique reflections	22695
R_merge_	0.05 (0.20)
*σ*	23 (6.5)

Refinement statistics	
Resolution range (Å)	44.2-1.5
Reflections/test set	22363/1135
R-factor	0.1657 (0.1819)
R_free_	0.1935 (0.2294)
No. of residues	127
No. of water molecules	189
No. of ligand atoms	4 sulfate ions
Average B-factor (Å^2^)	13.39
*r.m.s.d. from ideal*	
Bond lengths (Å)	0.006
Bond angles (deg.)	1.070
*Ramachandran statistics*	
Most favoured (%)	93.5
Additionally allowed regions (%)	6.5

### Structural comparison of CD4 binding site antibodies

D7 was shown to interfere with CD4 and b12 binding on gp120 and exerts a decent neutralization profile of primary HIV-1 clade B and C isolates [35]. Its neutralization capacity is similar to that of b12, although b12 is more potent and both antibodies neutralize a different spectrum of viruses [35]. MAb b12 employs only the heavy chain to interact with gp120, indicating that a heavy chain only antibody such as the D7 V_HH_ could mimic its interaction with gp120. All three b12 heavy chain CDRs contact gp120, notably mostly its outer domain [14]. The overlap between the b12 and the CD4 buried surfaces are considerable and both modes of interaction employ aromatic residues to fill a hydrophobic pocket on gp120 (b12 Tyr^53^ of CDR2 and CD4 Phe^43^) [13], [14]. Three other crystal structures of CD4 binding site mAbs are known, namely those of unliganded F105 and m18 Fabs [40], [41] and F105 and b13 in complex with gp120 [15]. F105, b13 and m18 interfere with CD4 binding on gp120, neutralize several HIV-1 strains but exert lower potency in neutralization of primary isolates as compared to b12 [15], [31], [42]–[44]. The structures of gp120 in complex with F105 and b13 show that it does not suffice to occupy the CD4 binding site in order to exert broad neutralizing activity since the orientation of gp120 recognition is most likely not compatible with binding to the trimeric Env present on virions [15].

**Figure 3:**
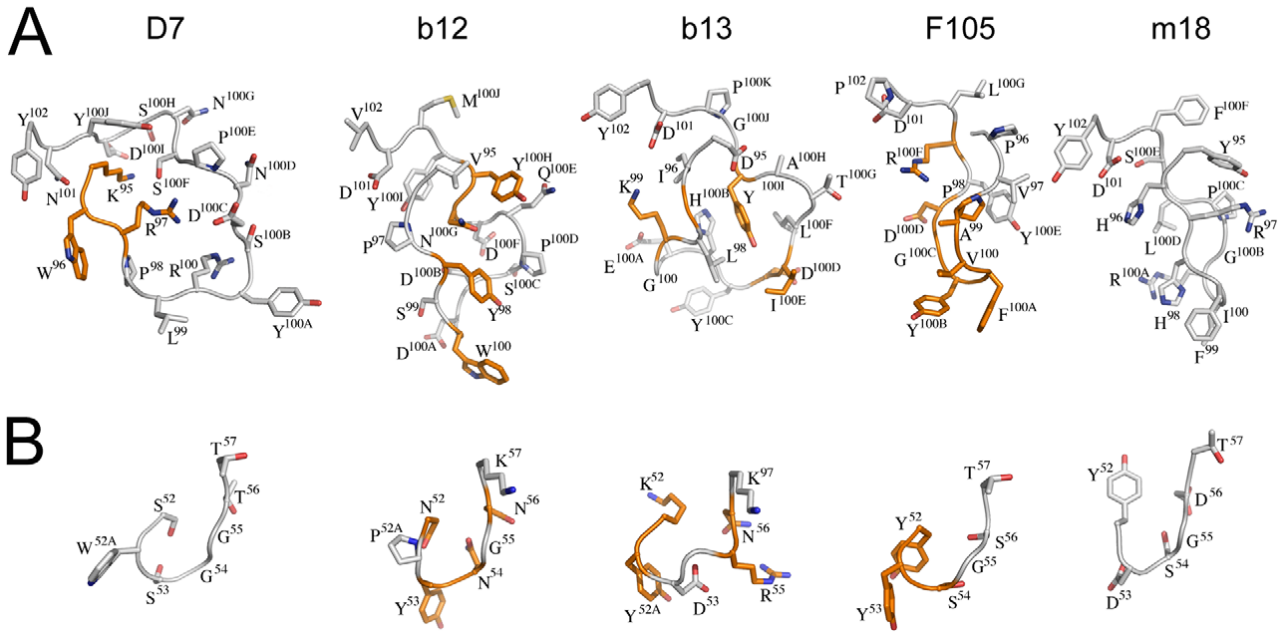
Comparison of CDR2 and CDR3 from D7, b12, b13, F105 and m18 indicates different modes of gp120 interaction. The Cα atoms of the heavy chain variable domains (b12 pdb code 2NY7; F105 pdb code 3HI1; b13 pdb code 3IDX; m18 pdb code 2AJ3) were superimposed and the CDR H2 and H3 are represented in the same orientation. Amino acids are labelled using the one letter code for clarity. (**A**) All CDR3 loops expose aromatic residues at their apex. (**B**) The CDR2 of D7 varies from CDR H2 of b12 indicating a different mode of gp120 interaction. Residues implicated in gp120 interaction are highlighted in orange.

CD4 binding site antibodies b12, b13, F105 and m18 display a similar architecture of their CDR3 heavy chains with aromatic residues positioned at the apex of their CDR3 (Figure 3A). This feature led originally to the proposal that this class of antibodies employs aromatic CDR residues to fill the gp120 pocket that is occupied by Phe^43^ in the CD4- bound state [41], [45]. Although b12 and b13 point their CDR2 residue Tyr^53^ and Tyr^52A^, respectively, towards the CD4 binding pocket [14], mAb F105 employs CDR3 Phe^100A^ and Tyr^100B^ within the CD4 binding site [15].

Sequence alignment of D7 with the heavy chain sequences of b12, b13, F105 and m18 reveals aromatic residues within CDR2 and CDR3 (Figure 2). Comparison of the CDR2 loops shows Tyr^53^ at the apex of F105 CDR2, no aromatic residue at the apex of m18 CDR2 and Tyr^53^ and Tyr^52a^ at the apex of b12 and b13 CDR2. The CDR2 of D7 contains Trp^52A^, which is however not solvent accessible. Instead D7 Trp^52A^ is involved in CDR1 stabilization as observed in another V_HH_ structure [36] (Figure 3B). It is thus unlikely that D7 CDR2 plays a prominent role in gp120 interaction as observed for CDR2 from b12 and b13.

We thus focused our analysis on the CDR3 region as a potential gp120 interaction site. Part of this loop region is highly mobile in the absence of ligand and thus suitable for an induced fit conformation. CDR3 of D7 is tilted 40° towards CDR2 compared to the orientation of the CDR H2/H3 of the other antibodies, whereas the apex of CDR3 is also built by an aromatic residue (Tyr^100A^) like in b12 (Trp^100^), F105 (Phe^100A^ and Tyr^100B^), b13 (Tyr^100C^) and m18 (Phe^99^). Notably, b12 employs a number of CDR H3 residues to contact gp120 including Trp^100^ (Figure 3B). However, despite the fact of aromatic residues at the tip of all CDR3 loops, no significant structural homology between the CDR loops can be observed underlining the differences in mode of gp120 interaction.

### Mutational analysis of D7 binding to gp120

We performed mutational analysis within CDR3 to substantiate the role of CDR3 in gp120 interaction by determining the affinity of the D7 mutants in comparison to the wild type using surface plasmon resonance. Mutagenesis of solvent exposed CDR3 residues Lys^95^Ser, Trp^96^Ala and Leu^99^Ala (Figures 2 and 3A; table 2) decreased the affinity of D7 to gp120 (IIIB) significantly to 377, 27 and 69 nM, respectively, compared to 2.9 nM of wild type D7 (table 3). Note that the affinity of D7 to gp120 determined here is ∼30 times lower than the previously reported K_D_ of 0.097 nM [35]. In contrast, mutagenesis of solvent exposed residues Asp^100C^, Asn^100D^ and the double mutant Asn^101^Tyr Tyr^102^Asp (table 2) enhanced the affinity to 0.84, 0.51 and 0.18 nM (table 3). Notably, the double mutation at position 101 and 102 to Tyr and Asp restores the CDR3 sequence of V_HH_ A12 (Figure 2), which has broader neutralization potency than D7 [35]. Mutagenesis of Tyr^100A^ located at the tip of CDR3 (Figure 3A) has no significant effect on binding (table 3). The moderate increase of affinity by mutagenesis of Asp^100C^ and Asn^100D^ might reflect a decrease of the rigidity of the loop since loop stabilizing interactions are affected (table 3 and Figure 1B). This could lead to an improved induced fit upon gp120 binding facilitated by an increased CDR flexibility. The positive effect of the double mutation of Asn^101^Tyr^102^ on interaction together with their close location next to Trp^96^ and Lys^95^ suggest that they affect binding directly. Finally, the strong decrease in affinity upon mutagenesis of residues Lys^95^, Trp^96^ and Leu^99^ indicate that these residues make crucial contributions to gp120 interaction. The decrease in affinity is mainly due to an increase of the dissociation rate of the D7 mutants (table 3), which suggests a change of interaction between the CDR3 loop and gp120 but no major change of loop conformation. Together, these data implicate that V_HH_ D7 interacts via CDR3 with gp120 and identify Lys^95^, Trp^96^ and Leu^99^ as key residues for this interaction. Both Lys^95^ and Trp^96^ at the beginning of CDR3 are solvent exposed while Leu^99^ is part of the disordered tip of CDR3, which adds to the conformational freedom of D7 interaction with gp120 (Figure 3A). All three residues are conserved in V_HH_ A12 suggesting that it will utilize its CDR3 to contact gp120 in a similar way (Figure 2). A12 differs only at positions 100D (Tyr instead of Asn) as well as 101 and 102 (Asn, Tyr instead of Tyr, Asp). Further sequence differences that may account for the higher neutralization potency of A12 include CDR1 residues (Thr^28^ is changed to Ile^28^ and His^32^ to Tyr^32^ in A12) and CDR2 residue Asn^58^ (Asp^58^ in A12).

**Table 2:** Solvent accessible areas of CDR3 D7 residues.

residue	accessible surface area (Å^2^)
Lys^95^	36.56
Trp^96^	136.34
Leu^99^	179.13
Tyr^100A^	220.36
Asp^100C^	47.52
Asn^100D^	80.47
Asn^101^	80.56
Tyr^102^	52.65

**Table 3:** Binding affinities of D7 wild type and D7 mutants to gp120 (IIIB).

mutation	k_a_ (10^5^ M^−1^ s^−1^)	k_d_ (10^−4^ s^−1^)	K_D_ (nM)
wildtype	1.55±1.15	5.51±1.47	2.91±0.87
Lys^95^→Ser	0.13±0.10	23.6±4.90	377±276
Trp^96^→Ala	1.46±0.46	31.3±17.1	27.9±20.5
Leu^99^→Ala	0.14±0.12	50.0±29.1	69.9±14.2
Tyr^100A^→Ala	2.33±0.15	3.59±0.08	1.55±0.07
Asp^100C^→Ala	2.36±0.13	1.99±0.38	0.84±0.12
Asn^100D^→Ala	2.52±0.06	1.28±0.03	0.51±0.02
Asn^101^→Tyr/Tyr^102^→Asp	1.87±0.08	0.33±0.27	0.18±0.15

**Figure 4:**
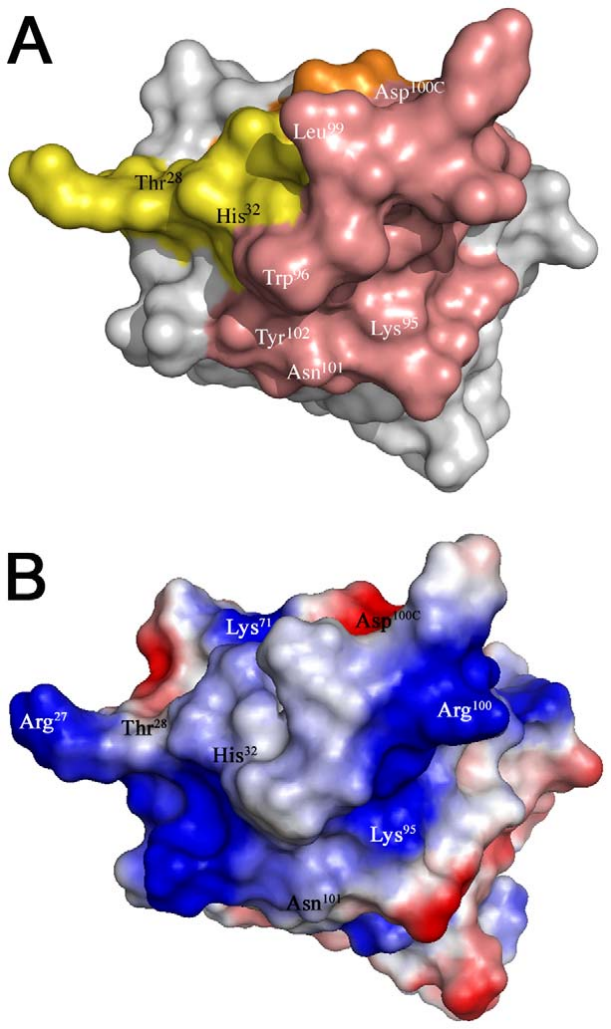
Surface representation of the CDRs. (A) Surface representation of D7 revealing the gp120 docking interface based on gp120 binding and HIV-1 neutralization results. The CDRs are coloured as in figure 1. CDR3 residues affecting gp120 interaction and HIV-1 IIIB neutralization are indicated in white and residue differences between D7 and A12 are labelled in black. (B) Electrostatic potential map of the surface generated by the CDRs.

In order to confirm the role of CDR3 and the correlation of gp120 interaction and neutralization, wild type D7 and mutants were tested in a TZM-b1 neutralization assay against HIV-1 IIIB. Comparison of the IC50 values reinforces the importance of CDR3. Mutations that lead to decreased gp120 interaction, namely Ala mutations of Lys^95^ and Trp^96^, showed a 100-fold increase in IC50, and Leu^99^ revealed a 10-fold increase in IC50 as compared to D7 wild type (table 4). In contrast, mutation of Tyr^100A^ and Asp^100C^ show a modest decrease in the IC50 (table 4) consistent with a slightly increased affinity for gp120 (table 3). The largest positive effect was observed for the double mutant Asn^101^Tyr^102^, which shows a ∼10-fold increase in affinity (table 3) and a reduction of the IC50 value by a factor of 5 (table 4). This underlines the important role of CDR3 (Figure 4A) for neutralization of HIV-1. Since the CD4 binding site on gp120 is negatively charged [13] CDR3 could provide some complementary basic charge for interaction (Figure 4B). Together these findings indicate that the differences related to CDR3 constitute an important factor accounting for the broader neutralization profile of A12 compared to D7 [35] since both CDR1 and CDR2 are almost identical (Figure 2). It is thus possible that C8 employs different structural principles for gp120 interaction and neutralization [35].

**Table 4:** IC_50_ values of V_HH_ D7 against HIV-1 IIIB in TZM-bl cells.

	IC_50_ (µg/ml)	IC_50_ (µg/ml)	IC_50_ (µg/ml)
mutation	Exp1*	Exp2*	Average**
wildtype	0.066	0.041	0.054
Lys^95^→Ser	3.426	2.641	3.0
Trp^96^→Ala	2.717	2.428	2.6
Leu^99^→Ala	0.817	0.560	0.69
Tyr^100A^→Ala	0.028	0.018	0.023
Asp^100C^→Ala	0.40	0.014	0.027
Asn^100D^→Ala	ND	ND	ND
Asn^101^→Tyr/Tyr^102^→Asp	0.017	0.005	0.011

* ) Experiments were carried out in duplicate wells.

### Conclusions

The conformation of the primary receptor binding site of HIV-1 gp120 reveals a hydrophobic pocket which is the target for Phe^43^ of the natural receptor CD4 [13] and overlaps with the binding sites of neutralizing antibodies b12 [14], b13 and F105 [15]. The structural and mutational data presented here show that D7 does not expose an aromatic residue at its CDR2 and that Tyr^100A^ at the apex of CDR3 does not play a key role in gp120 interaction and HIV-1 IIIB neutralization. Thus D7 might employ different structural principles than b12 to interact with gp120. This is further supported by preliminary results on the D7 interaction with HIV-1 envelope proteins gp140_CN54_, gp140_UG37_, gp120_IIIB_, gp120_YU2_ and its modified variant gp120Ds2, in which an additional S-S (109-428) bridge was introduced, thus closing the cavity below the bridging sheet [14]. V_HH_ D7 binds to gp120_IIIB_, gp140_UG37_ and gp120_YU2_ but is unable to interact with gp120Ds2 strongly indicating that D7 does not bind to the outer domain, as b12 does (A. Szynol personal communication). This is further supported by antibody recognition of a gp120 escape mutant. Whereas gp120 mutation G366E results in impaired binding of sCD4 and b12, the binding of D7 was not effected (A. McKnight, personal communication), further corroborating differences in gp120 interaction of b12 and D7. Although we identified Trp^96^ as a key residue for gp120 interaction and neutralization its position and limited extension form the core structure albeit its solvent exposure (table 4) does not conclusively indicate how it could reach into the hydrophobic CD4 binding pocket on gp120. Thus CD4 binding site antibodies might be broadly neutralizing without closely mimicking important molecular details of the CD4-gp120 interaction. Although the structure establishes a firm role for CDR3 and its flexible anchoring in interaction and neutralization activity, structural analysis of gp120 in complex with D7 is required to fully understand the conformational flexibility of both gp120 and D7 in order to exploit this knowledge for a rational vaccine design.

## Materials and Methods

### D7 purification, crystallization and structure solution

*S. cerevisiae* strain VWk18gal1 (CEN-PK102-3A, MATa, leu2-3, ura3, gal1::URA3, MAL-8, MAL3, SUC3) was used for the fermentative production of D7. The V_HH_ D7 gene contains the following amino acid substitutions compared to wild type D7 [35] in framework residues Val5Gln, Ala11Val, Ala61Val, Ala68Asp and Ser79Tyr, which occur naturally in other known V_HH_ structures [36]. The gene was integrated into the *S. cerevisiae* genome in the rDNA locus [46] and grown as described [47]. V_HH_ D7 was purified from the supernatant using Ni^2+^-affinity chromatography in PBS. A final size exclusion chromatography was performed on a Superdex 200 (GE Healthcare) in a buffer containing 20 mM HEPES pH 7, 0.1 M NaCl. V_HH_ D7 was crystallized using the hanging drop vapor diffusion method at room temperature. Crystals formed by mixing D7 (at 5 mg/ml) with an equal volume of 100 mM sodium cacodylate pH 6, 20 mM magnesium acetate, 1.7 M ammonium sulphate and 19% glycerol. Crystals were flash frozen in liquid nitrogen using 30% glycerol as cryo-protectant. A native dataset was collected to a resolution of 1.5 Å at ESRF beam line (Grenoble, France) BM14. The dataset was processed with MOSFLM [48] and SCALA [49], [50]. The crystals belong to space group P2_1_2_1_2_1_ with unit cell dimensions of a = 37.37 Å, b = 62.18 Å, c = 62.74 Å containing 1 molecule in the asymmetric unit.

The structure was solved by molecular replacement using the program PHASER [51] and the V_HH_ structure pdb code 1HCV as a search model. The initial model was built using ARPWARP [52] and completed by several cycles of manual rebuilding in COOT [53] and refinement in REFMAC [54] and PHENIX [55] to R_work_/R_free_ values of 0.1657/0.1935. The final model contains 127 residues, 189 water molecules and 4 sulfates (see table 1). All molecular graphics figures were generated with PYMOL (W Delano; http://www.pymol.org/). Coordinates and structure factors of D7 have been deposited in the Protein Data Bank with accession number 2xa3.

### Mutation of V_HH_ D7 and Surface Plasmon Resonance analysis of D7 binding to gp120

Mutations of the V_HH_ D7 sequence [35] were generated by using the quick change mutagenesis kit (Stratagene). The mutations were verified by sequencing. Wild type and mutant V_HH_ D7 proteins were expressed in *E. coli* and purified following the protocol for wild type D7 purification [35].

Gp120 (IIIB) was coupled covalently to channel 2 of a CM5 chip (GE Healthcare) following the manufacturer's instructions. Briefly, the CM5 chip was activated with 50 µL EDC/NHS at a flow rate of 25 µl/min. Adsorption of 100 µl gp120 was carried out in a buffer containing 25 mM sodium acetate pH 5. Deactivation of the surface was achieved with 50 µl diethanolamine. Chanel 1 was treated similar but skipping the step of gp120 adsorption. All binding experiments were performed on a Biacore X (GE Healthcare) in a buffer containing 10 mM HEPES pH 7.5, 100 mM NaCl, 0.05% P20 at a flow rate of 30 µl/min. The association of 90 µl D7 or D7 mutants at concentrations of 25 and 100 nM was recorded for 3 min followed by a dissociation of 15 min. The CM5 chip was regenerated with a 20 µl pulse of 0.1 M glycine pH 2 at a flow rate of 50 µl. Data were evaluated with the BiaEvaluation software (GE Healthcare) using simultaneous fit of association and dissociation.

### HIV-1 neutralization in TZM-bl cells

HIV-1 neutralization in TZM-bl cells was evaluated using an assay developed previously [56], [57], [58]. Three-fold serial dilutions of V_HH_ (starting at 20 µg/ml) were prepared in growth medium (DMEM containing 10% FCS) in duplicate wells of opaque 96-well cell culture plates, in a total volume of 50 µl per well. Approximately 200 TCID50 of virus, in 50 µl of growth medium, was added to each well, and the plates were subsequently incubated at 37°C. After 1 hour of incubation, 1 ⋅ 10^4^ newly trypsinized TZM-bl cells in 100 µl of growth medium containing 30 µg/ml of DEAE-dextran (Sigma-Aldrich) were added to each well. For each plate, six wells containing cells and growth medium only, and six wells containing virus and cells only, were included. The neutralization activity of each V_HH_ was assayed in duplicate. The plates were then incubated at 37°C for 48 hours and detection of infection of TZM-bl cells was assayed by measuring luminescence production according to the manufacturer's instructions (Promega). Lysis of cells was allowed to occur for 2 minutes and luminescence (in relative light units; RLU) was then detected using a GloMax 96 Luminometer (Promega). Neutralization was measured as the reduction in RLU in test wells compared to virus control wells after subtraction of background luminescence. The lowest V_HH_ or antibody concentration required to give 50% reduction in RLU (IC_50_) was determined by fitting the data to a sigmoidal equation using the XLFit 4 software (IDBS).
